# Intra-articular platelet-rich plasma injection versus hydrocortisone with local anesthetic injections for temporo mandibular disorders

**DOI:** 10.6026/97320630018991

**Published:** 2022-10-31

**Authors:** Jayant Prakash, Dhaniram Talukder, Krunal Desai, Tarun K Singh, Reena Bagde, Gagandeep S Randhawa, Shambhavi Jha, Kapil Paiwal

**Affiliations:** 1 Department of Dentistry, Sadar Hospital, Muzaaffarpur-842001, Bihar, India; 2 Department of Prosthodontics and Crown and Bridge, DJ college of dental sciences and research, Modinagar, U.P, India; 3 NYU College of Dentistry, 345 E. 24th Street (corner of First Avenue), New York, NY 10010; 4 Department of Dentistry, AIIMS, Bathinda, India; 5 Department of Conservative Dentistry and Endodontics, Bharati Vidyapeeth Deemed to be University Dental college and Hospital, Sector 7CBD Belapur, Navi Mumbai, 400614, India; 6 Department of Oral and Maxillofacial Surgery, Ahmedabad Dental College and Hospital, Ahmedabad, India; 7 New Horizon Dental College and Research Institute, Sakri, Bilaspur (C. G) 495001, India; 8 Department of Oral Pathology and Microbiology, Daswani Dental College & Research Center, Kota,India

**Keywords:** Hydrocortisone with local anesthetic, Intra-articular injection, platelet-rich plasma injection, temporomandibular disorders

## Abstract

Disorders of temporomandibular joint (TMDs) are characterised by a variety of symptoms, including discomfort in the orofacial region, muscle tenderness, restricted jaw motion and noise at the joint. Additional neurological symptoms such as headaches, vertigo, heaviness, and altered vision may also coexist with TMDs. Because prostaglandin production is a critical mediator of inflammatory reaction and is inhibited by corticosteroids, they have anti-inflammatory effects. Platelet rich plasma popularly considered as PRP is a concentration of platelets and related growth factors that may have therapeutic effects by attracting, promoting, and differentiating cells as well as redesigning tissue. 64 joints totaling 40 individuals with temporomandibular joint problems were split into two categories (Category A and Category B). PRP was applied to category A's (36 joints of 20 patients) joints, while Group B's joints received hydrocortisone combined with local anaesthetic (28 joints of 20 patients). Patients were evaluated for tenderness, , maximum inter incisal opening (MIO) and clicking sound at TMJ prior to and following treatment at intervals of one week, one month and 6 months of the first week and third month. When there was comparison of outcomes in study participants receiving injections of platelet rich plasma and study participants receiving injections of hydrocortisone with local anesthetic then it was found that although both type of study participants got reduction in pain, increased opening of mouth and reduction in clicking sound however the difference between two groups was not significant statistically. There was no statistically significant difference between injections of platelet rich plasma and hydrocortisone with local anesthetic solution regarding outcomes in disorders of temporomandibular joint, however the results were slightly better in study participants receiving platelet rich plasma injections. This study demonstrated that while treating patients with TMJ issues, local anaesthesia combined with hydrocortisone as well as intra-articular injection of PRP help in reduction in pain, increase mouth opening, and minimize joint sound. Additionally, it was discovered that intra-articular injection of PRP was more successful in treating patients in this trial than local anaesthetic combined with hydrocortisone.

## Background:

Temporomandibular disorders are a group of pathological health conditions that affect the muscles involved in food mastication as well as related soft tissue components. They are consequently regarded as musculoskeletal disorders of the head and neck's masticatory assembly. TMDs are characterised by a variety of symptoms, including discomfort in the orofacial region, muscle tenderness, restricted jaw motion, noise at the joint, inhibited jaw function, alteration or diversion, rigidity, pain or lethargy in the muscles of the face, and locking brought on by muscle spasm.[1]Additional neurological symptoms such as headaches,vertigo, heaviness, and altered vision may also coexist with TMDs.In order to compare the effectiveness of PRP injections with injections of hydrocortisone using local anaesthetic in the conservative care of disorders of temporomandibular joint, we therefore started this double-blind, randomized study. [2] There are many factors that can predispose to, increase, or aggravate TMD, including muscular impulsivity, trauma, mental anguish,and malocclusion. Pain relief, the restoration of normal jaw function, and the maintenance of a normal way of life are the key objectives of treatment for all TMD patients. To treat these diseases,a variety of therapeutic techniques can be used, such as conservative therapy, behavioural therapy, physiotherapy, pharmacological therapy, and occlusal appliances. [3] Because prostaglandin production is a critical mediator of inflammatory reaction and is inhibited by corticosteroids, they have antiinflammatory effects. Platelet rich plasma popularly considered as PRP is a concentration of platelets and related growth factors that may have therapeutic effects by attracting, promoting, and differentiating cells as well as redesigning tissue. [4] PRP administration may have an inhibitory effect on specific proinflammatory mediators that could be detrimental to the early stages of healing, particularly through the reduction of interleukin1 production from activated macrophages in conjunction with the inducing actions on reparative cells. [5] PRP increases chondrogenic proliferation and the production of matrix molecules, which facilitates joint movement and preserves the chondral surface's overall structure. According to observations made in the available literature, the cannabinoid receptors CB1 and CB2 were found to be enhanced, which may be related to the analgesic effects of PRP. PRP may aid in the therapy of degenerative joint illnesses by lowering pain and enhancing joint sound, according to recent studies. [6] Corticosteroids are anti-inflammatory medications that are effective because they penetrate cells and bind to the glucocorticoid receptor. [7]The steroid and receptor complex enters the nucleus, binds with DNA in a certain order, and boosts the expression of genes that combat inflammation. In addition, corticosteroids stop the inflammatory mediator’s prostaglandins from being produced. However there have been evidence of serious adverse effects of corticosteroids. [8] In order to compare the effectiveness of PRP injections with injections of hydrocortisone using local anaesthetic in the conservative care of disorders of temporomandibular joint, we therefore started this double-blind, randomized study

## Methods and Materials:

64 joints totaling 40 individuals with temporomandibular joint problems were split into two categories (Category A and Category B). PRP was applied to category A's (36 joints of 20patients) joints, while Group B's joints received hydrocortisone combined with local anaesthetic (28 joints of 20 patients). The components of the injections used during arthroscopy were hidden from the patient as well as the clinician. Patients were evaluated for tenderness, , maximum inter incisal opening (MIO) and clicking sound at TMJ prior to and following treatment at intervals of one week, one month and 6 months of the first week and third month.

## Inclusion criteria:

Patients having a history disorder of temporomandibular joint,clicking at TMJ region, and pain in TMJ region when moving their jaws.

## Exclusion criteria:

Patients with head and neck malignancies, neurological disorders,severe anaemia, history of thrombocytopenia, history of connective tissue diseases, or inflammatory diseases

## Methods:

## Preparation of platelet-rich plasma:

The PRP specimens were processed in an aseptic manner. The first phase involved centrifuging ten ml of citrated blood of study participants at one thousand eighteen hundred rpm for fifteen minutes. To consolidate the concentration of platelets, the second phase involves centrifuging the plasma-rich layer removed during the first centrifugation, at 3500 rpm for ten minutes. Finally, PRP was gathered into a sanitized injector for injection.

## Operative technique:

A line was established from the center of the tragus region to the outside canthus to mark the preauricular zone, which was subsequently cleaned and made aseptic. The spot for the injection of PRP was designated on the line just 2 mm underneath and 10 mm forward to the tragus. A syringe with 20 gauge needle was used to inject 0.6 cc of PRP through into TMJ. Following injection, the patient was instructed to both close and open their mouth repeatedly few times to make certain that PRP was distributed evenly throughout the TMJ's superior joint region. 0.5 ml of systematic hydrocortisone and 1 ml of local anesthetic solution was injected using the same anatomical tracing. The participant's jaws should be parted widely throughout the process. The patient is instructed to do sideways and forward motions of mandible after the injection. It was instructed to apply ice to the injection site for a short period of time.

## Assessment of treatment outcomes:

TMJ tenderness was evaluated using a visual analogue scale, which ranges from 0 to 10, with 0 representing no pain and 10 representing the most excruciating pain possible. Maximum interincisal opening considered as MIO was measured using a vernier calliper set at millimetres. The preauricular region was covered with a stethoscope to evaluate TMJ clicking noise. The same researcher conducted these assessments again during the follow-up visits one week, one month, three months, and six months after the operations. A total of 8 patients - 4 from each group - were not followed. Finally, statistical analysis was performed on 44 patients (22 in each group).

## Statistical analysis:

The industry-standard SPSS program version 18 was used to conduct the statistical analysis. The T test was used to compare the groups' pain levels, maximum interincisal openness, and TMJ clicking. The variables within the groups were compared over various time periods using Repeated Measure ANOVA. For all statistical analyses, 0.05 was the threshold for significance.

## Results:

In this study VAS score at baseline in study participants receiving injections of platelet rich plasma was 7.81±1.27. It got reduced to 6.42±1.73 after one week of administration of injections. It further decreased to 4.29±1.61. After three months of therapy the VAS score was 2.47±1.84. Finally it was 1.11±0.86 at 6 month of follow up. When there was analysis of maximum interincisal opening of mouth then it was found to be 34.74±6.71 mm at baseline. It improved to 36.13±6.86 mm at one week follow up. It become 38.6 ±3.12 mm at one month of follow up. The maximum mouth opening was 40.5 ±1.84mm at 3 months of follow up. The maximum mouth opening at 6 months of follow up was 39.86±2.86 mm. There was assessment of clicking sound at TMJ in study participants receiving injections of platelet rich plasma. It was 23±0.43 at baseline, 21±0.46 at one week follow up, 16±0.73 at one month follow up, 12±0.72 at 3 month follow up, 6±0.4 at 6 months follow up. The findings were statistically significant with p ≤ 0.05 with reduction of pain, improved mouth opening, decrease in clicking sound at TMJ. (Table 1 - see PDF)

VAS score at baseline in study participants receiving injections of hydrocortisone with local anesthetic solution was 8.92 ±1.27. It got reduced to 7.21±1.52 after one week of administration of injections. It further decreased to 6.10±2.01 at one month follow up. After three months of therapy the VAS score was 4.96±1.73. Finally it was 2.00±0.75 at 6 month of follow up. When there was analysis of maximum interincisal opening of mouth then it was found to be 34.61±7.02 mm at baseline. It improved to 35.15±5.75 mm at one week follow up. It becomes 36.52±4.03 mm at one month of follow up. The maximum mouth opening was 38.31±2.73 mm at 3 months of follow up. The maximum mouth opening at 6 months of follow up was 38.97±2.86 mm. There was assessment of clicking sound at TMJ in study participants receiving injections of platelet rich plasma. It was 24±0.12 at baseline, 23±0.21 at one week follow up,17±0.47 at one month follow up, 13±0.51 at 3 month follow up, 7 ±0.4 at 6 months follow up. The findings were statistically significant with p ≤ 0.05 with reduction of pain, improved mouth opening, decrease in clicking sound at TMJ. (Table 2 - see PDF)

When there was comparison of outcomes in study participants receiving injections of platelet rich plasma and study participants receiving injections of hydrocortisone with local anesthetic then it was found that although both type of study participants got reduction in pain, increased opening of mouth and reduction in clicking sound however the difference between two groups was not significant statistically. (Table 3) There was no statistically significant difference between injections of platelet rich plasma and hydrocortisone with local anesthetic solution regarding outcomes in disorders of temporomandibular joint; however the results were slightly better in study participants receiving platelet rich plasma injections.

## Discussion:

PRP is a dense accumulation of platelets and related growth factors (GFs) that is drawn from the blood of a person. PRP has been therapeutically used for a variety of procedures, including softtissue ulcer management, spinal fusion condition, and surgery in oral as well as maxillofacial region, aesthetic plastic surgery, and surgery of periodontal tissues. When PRP is applied, substances produced from platelet alpha granules and other chemical mediators flow into the joint area's milieu.[9] The elevated platelet and GF concentration mimics the early phase of the inflammatory process, which is characterised by neutrophil cells, monocyte cells, and macrophage cells migration to the injury location. Process of neo vascularization, process of fibroblast proliferation, and additional inflammatory cell mobilization are all mediated by mediators and cytokines. [10] Local PRP administration might exert an inhibitory influence on particular pro-inflammatory mediators that may be harmful to the initial stages of healing, particularly through the reduction of interleukin-1 production from activated macrophages, in conjunction to the inducive actions on reparative cells. PRP helps to preserve the overall structure of the chondral surface as well as consequently makes joint movement easier by increasing chondrogenic multiplication and the generation of matrix molecules. [11] The cannabinoid receptor like CB1 and CB2 were found to be increased, which may be associated to the analgesic benefits of PRP, as observed in the available literature. Recent research has indicated that PRP may help in the management of degenerative joint diseases by reducing pain and improving joint sound. [12] In this study VAS score at baseline in study participants receiving injections of platelet rich plasma was 7.81±1.27. It got reduced to 6.42±1.73 after one week of administration of injections. It further decreased to 4.29±1.61. After three months of therapy the VAS score was 2.47±1.84. Finally it was 1.11±0.86 at 6 month of follow up. When there was analysis of maximum interincisal opening of mouth then it was found to be 34.74±6.71 mm at baseline. It improved to 36.13±6.86 mm at one week follow up. It become 38.6 ±3.12 mm at one month of follow up. The maximum mouth opening was 40.5 ±1.84mm at 3 months of follow up. The maximum mouth opening at 6 months of follow up was 39.86±2.86 mm. There was assessment of clicking sound at TMJ in study participants receiving injections of platelet rich plasma. It was 23±0.43 at baseline, 21±0.46 at one week follow up,16±0.73 at one month follow up, 12±0.72 at 3 month follow up, 6±0.4 at 6 months follow up. The findings were statistically significant with p ≤ 0.05 with reduction of pain, improved mouth opening, decrease in clicking sound at TMJ. The term "temporomandibular disorders" (TMDs) refers to a group of general health-related and dental related disorders that impact the muscles contributing in mastication of food as well as associated soft tissue elements. They are therefore considered as musculoskeletal disease of masticatory assembly of head and neck. [13] Pain in the orofacial region, tenderness of muscle , restricted in motion of jaws, noise at the joint, inhibited function of jaws, alteration or diversion, rigidity, pain or lethargy in the muscles of face, and locking brought on by muscle spasm are just a few of the signs and symptoms that define TMDs. Besides, TMDs may also be accompanied by additional neurological symptoms as headaches, vertigo, heaviness, and vision abnormalities. [14] Numerous predisposing, amplifying, and aggravating circumstances among which muscle impulsivity, trauma, mental turmoil, and malocclusion all contribute to the aggravation of TMD. The primary goals of treatment for all TMD patients are pain relief, the return of normal jaw function, and the maintenance of a normal way of life. [16]Numerous therapeutic modalities, including conservative therapy, behavioral therapy, physiotherapy, pharmaceutical therapy, and occlusal appliances can be used to manage these disorders. [15] More effective anti-inflammatory drugs include corticosteroids, which work by entering cells and attaching to the glucocorticoid receptor. [17] Complex of steroid and receptor enters the nucleus, interacts with DNA in a precise sequence, and increases the expression of genes that fight inflammation. Additionally, corticosteroids prevent the production of prostaglandins, which are inflammatory mediators. [18] VAS score at baseline in study participants receiving injections of hydrocortisone with local anesthetic solution was 8.92 ±1.27. It got reduced to 7.21±1.52 after one week of administration of injections. It further decreased to 6.10±2.01 at one month follow up. After three months of therapy the VAS score was 4.96±1.73. Finally it was 2.00±0.75 at 6 month of follow up. When there was analysis of maximum interincisal opening of mouth then it was found to be 34.61±7.02 mm at baseline. It improved to 35.15±5.75 mm at one week follow up. It becomes 36.52±4.03 mm at one month of follow up. The maximum mouth opening was 38.31±2.73 mm at 3 months of follow up. The maximum mouth opening at 6 months of follow up was 38.97±2.86 mm. There was assessment of clicking sound at TMJ in study participants receiving injections of platelet rich plasma. It was 24±0.12 at baseline, 23±0.21 at one week follow up, 17±0.47 at one month follow up, 13±0.51 at 3 month follow up, 7 ±0.4 at 6 months follow up. The findings were statistically significant with p ≤ 0.05 with reduction of pain, improved mouth opening, decrease in clicking sound at TMJ. The knee joint has been documented to be negatively affected by corticosteroids injected into intra articular areas. These side effects include infectious arthritis, condition of "flare," after injections, atrophy of local tissue, rupture of tendon, destruction of cartilage, flushing, and elevated blood sugar levels. [19] These negative consequences don't happen very often. We began this trial of PRP injections in intraarticular PRP in contrast to corticosteroid with extended acting injections in an effort to lessen these drawbacks of corticosteroids. Furthermore, PRP produced remarkably positive benefits. [20] In this study when there was comparison of outcomes in study participants receiving injections of platelet rich plasma and study participants receiving injections of hydrocortisone with local anesthetic then it was found that although both type of study participants got reduction in pain, increased opening of mouth and reduction in clicking sound however the difference between two groups was not significant statistically. There was no statistically significant difference between injections of platelet rich plasma and hydrocortisone with local anesthetic solution regarding outcomes in disorders of temporomandibular joint; however the results were slightly better in study participants receiving platelet rich plasma injections. Among the most frequently utilized joints within the human body is the temporomandibular joint. The condyle of mandible and articular facets of temporal bone combine to produce the composite articulation known as the TMJ. [21] A thick layer of articular fibrocartilage covers both articulating surfaces. Both condyle of mandible articulates with a sizable portion of the anatomic entity preglenoid plane, anatomic entity articular eminence, and anatomic entity articular fossa of the temporal bone. Because the condyle moves in anterior direction along with the articular eminence & it rotates inside the fossa, the TMJ functions differently from other joints. The lower jaw can have significantly greater maximal incisal openness than would be feasible with rotation of condyle alone due to the mandibular condyle additional capacity to translate. Consequently, gynglimodiarthrodial, a blend of the phrases ginglymoid (meaning rotation) and arthroidial (meaning translation), is the name given to the joint. [22] Muscular TMDs and articular TMDs are the two broad categories of TMDs. Due to the potential overlap and similarity of muscle illnesses and articular disorders; it can be challenging to distinguish between the two. Condition of myalgia, condition of myospasm, condition of splinting, and condition of fibrosis or contracture are examples of myogenic diseases. Condition of synovitis or condition of capsulitis, condition of joint effusion, condition of trauma or fracture, condition of internal derangement, condition of arthritis, and tumour are examples of articular diseases. [23] Since the disturbed joint will keep attempting to function, internal derangement disorders worsen over time. Variation in collagen component, extracellular matrix component, macromolecules components, and proteoglycans components lead to TMD by altering the structural integrity of meniscus and subchondral bone. [24] Platelet-rich plasma (PRP)may be beneficial in the management of joint degenerative disorders, according to emerging research. In a research with prolonged follow-up statistics, Hegab AF et al. compared the application of PRP versus hyaluronic acid in the management of temporomandibular joint osteoarthritis. [21] To one of two trial groups that were given either PRP or HA, patients were randomized. Maximum non-assisted opening of mouth, joint noises, and pain index ratings were the outcome indicators. Age and gender of the patients were examined in connection with the outcomes of other factors. In view of the reappearance of pain and joint noises at six months and twelve months, the study participants of PRP group performed better than the Hyaluronic acid group. TMJ injections containing a local anaesthetic and a corticosteroid may be a viable first-line treatment option for people with restricted mouth opening, however this is debatable. In order to ascertain the efficacy of injections of corticosteroids and local anaesthetic solutions in TMJ in patients with locked jaws i.e. anterior disc displacement without reduction, Samiee et al. undertook a study. As an alternate first-line therapy approach for locked jaw, injection of corticosteroid along with local anaesthesia in TMJ is suitable. [6] The use of three-dimensional scaffolds, such as hydrogels, for the delivery of cells and drugs has grown to be a prominent area of study in tissue engineering. Using a rabbit model, Lee HR et al looked examined the ability of PRP along with chondrocyte or hydrogel hybrid scaffold to repair damaged articular cartilage. [20] Hydrogels were used to cultivate fresh secluded chondrocytes of joints. In a time-dependent strategy, the hydrogel significantly enhanced cellular survivability and their development. The hydrogels also decreased the production of type II collagen biostructure, aggrecan biostructure, and SOX-9 biostructure. When compared to both control as well as PRP-free hydrogels, PRPcontaining hydrogels immediately increased the mRNA levels of the cannabinoid receptors CB1 as well as CB2. With the development of cartilage structure and the perichondrium structure in the combination of hydrogel + chondrocytes+PRP, osteochondral defects recovered more quickly. In connection to the emulsification density and bioactive elements like PRP, hydrogel may offer a favourable environment for the multiplication and differentiation of joint chondrocytes, improving cartilage regeneration. [12, 13] TMD patients have involved the intraarticular application of medications such as morphine pharmacological agent, corticosteroids pharmacological agents, longer acting local anaesthetic pharmacological agents, lower as well as higher molecular hyaluronic acid, with varying degrees of efficacy. From one to many sessions, various medication administration regimens have been attempted. [14, 17] One such study demonstrated no statistically significant difference in the treatment outcome characteristics across the six distinct intraarticular medication injection regimens. Brennan discovered that 90% of patients with disorders of temporomandibular experienced pain reduction even one year after receiving intraarticular morphine infusion following arthrocentesis. Along with the application of morphine for longterm pain management, they advised arthrocentesis as an efficient and minimally invasive treatment for TMJ pain relief. [19, 21] In studies comparing hyaluronic acid and PRP for TMJ osteoarthritis,PRP performed better at prolonged follow-up. When PRP was utilised for intraarticular TMJ injection, Moon et al. discovered considerable reduction in TMJ discomfort and increased maximum interincisal gap, but the reduction in TMJ noises was not statistically meaningful. Following intra-articular injections of platelet-rich plasma through the TMJ [22] Pihut et al. found that patients with disfunction of temporomandibular joint experienced less intense pain. [23] PRP injections for management of osteoarthritis of temporomandibular are helpful for pain reduction and TMJ function improvement, according to Zhao et al meta analysis. Additionally, it was found that the representative sample and gender distribution were significant factors in predicting PRP's effectiveness.[24] The primary care doctors will be able to identify resistant patients of the condition of disorders of temporomandibular joint with the aid of this study. Additionally, they can assist the patients in understanding that a platelet-rich plasma injection into the temporomandibular joint can effectively treat persistent TMD.

## Conclusions:

This study demonstrated that while treating patients with TMJ issues, local anaesthesia combined with hydrocortisone as well as intra-articular injection of PRP help in reduction in pain, increase mouth opening, and minimize joint sound. Additionally, it was discovered that intra-articular injection of PRP was more successful in treating patients in this trial than local anaesthetic combined with hydrocortisone.

## Author's contribution:

Jayant Prakash: Contributed to conception, design, data acquisition and interpretation, drafted and critically revised the manuscript. Dhaniram Talukder: Contributed to conception, design, data acquisition and interpretation, performed all statistical analyses, drafted and critically revised the manuscript. Krunal Desai:Contributed to conception, design, and critically revised the manuscript. Tarun K Singh: Contributed to conception, design, and critically revised the manuscript. Reena Bagde: Contributed to conception, design, and critically revised the manuscript. Gagandeep S Randhawa: Contributed to conception, design, and critically revised the manuscript. Shambhavi Jha: Contributed to conception, design, and critically revised the manuscript. Kapil Paiwal: Contributed to conception, design, and critically revised the manuscript. All authors gave their final approval and agree to be accountable for all aspects of the work.
